# Switching modes in corticogenesis: mechanisms of neuronal subtype transitions and integration in the cerebral cortex

**DOI:** 10.3389/fnins.2015.00274

**Published:** 2015-08-11

**Authors:** Kenichi Toma, Carina Hanashima

**Affiliations:** ^1^Laboratory for Neocortical Development, RIKEN Center for Developmental BiologyKobe, Japan; ^2^Department of Biology, Graduate School of Science, Kobe UniversityKobe, Japan

**Keywords:** neocortex, cell fate specification, neurogenesis, Cajal-Retzius cell, subplate, layer

## Abstract

Information processing in the cerebral cortex requires the activation of diverse neurons across layers and columns, which are established through the coordinated production of distinct neuronal subtypes and their placement along the three-dimensional axis. Over recent years, our knowledge of the regulatory mechanisms of the specification and integration of neuronal subtypes in the cerebral cortex has progressed rapidly. In this review, we address how the unique cytoarchitecture of the neocortex is established from a limited number of progenitors featuring neuronal identity transitions during development. We further illuminate the molecular mechanisms of the subtype-specific integration of these neurons into the cerebral cortex along the radial and tangential axis, and we discuss these key features to exemplify how neocortical circuit formation accomplishes economical connectivity while maintaining plasticity and evolvability to adapt to environmental changes.

## Introduction

Information processing in the neocortex relies on a highly ordered cytoarchitecture and its neuronal assembly to serve higher cognitive functions, such as perceptions, voluntary movements, and language. Neocortical neurons are organized into six major layers along the radial axis, which are further modified tangentially across areal and columnar subdivisions. These laminar and tangential organizations are key aspects of the cerebral cortex and are conserved among mammalian species, and they are thought to underlie the increase in neuronal numbers and expansion of the neocortex during evolution (Rakic, 2009). While the distinguishing feature of cellular organization of the cerebral cortex was acknowledged over a century ago (Meynert, 1868; Brodmann, 1909), the molecular mechanisms underlying the development and assembly of each neuronal component of the neocortex have rapidly begun to unravel over the past decade.

A major challenge in neocortical development is to efficiently recruit diverse cell types into its circuitry through the cost-effective production and wiring of individual neuronal elements. As dendrites and axons occupy the dominant fraction of the neocortical volume (Braitenberg and Schuz, 1998), minimizing neuronal process length in cortical network while maximizing their coverage is a key strategy in recruiting diverse neuron types in a restricted cortical capacity. In theory, this aim could be achieved through the reduction of molecular and wiring components while optimizing their networks; however, in a broader context, such effective topology and energy-saving construction is ideally adaptable to environmental and evolutionary changes.

For this purpose, the construction of the neocortical circuit becomes a highly dynamic process, which involves two fundamental steps that regulate the temporal and spatial behavior of cells during the progenitor and postmitotic stages. First, diverse neocortical neurons are generated from a restricted pool of progenitor cells within the ventricular and subventricular zones (VZ and SVZ), which differ in their connectivity, dendritic morphology, and molecular character. Second, the movement of cells from their place of birth to their final destination is an essential step to recruit these diverse neurons into the circuit and accommodate massive numbers of neurons within a restricted head volume.

In early development, the cerebral cortex starts from a simple neuroepithelial sheet at the anterior neural tube. This sheet gives rise to two major cell types of the neocortex, neurons and glia. The former are further classified into glutamatergic projection neurons and GABA (γ-aminobutyric acid)-ergic interneurons, which participate directly in the cortical circuit through the excitation and inhibition of distal and proximal target neurons, respectively. The glia, in turn, which include astrocytes and oligodendrocytes, play pleiotropic roles in shaping the cortical circuit by modulating its activity (Muller and Best, 1989; Chung et al., 2013). At a glance, the neocortical cytoarchitecture can be defined by its glutamatergic neuron components (Brodmann, 1909). In this review, we focus exclusively on the glutamatergic subtypes of the neocortex and reveal the organizing principles of the neocortical circuit through understanding the mechanisms by which neuronal subtype identity and integration are instructed in the cerebral cortex.

## Key elements of the neocortical scaffold

### Radial glial cells and transition from symmetric to asymmetric cell divisions

Genetic fate-mapping and loss-of-function studies have shown that neocortical excitatory neurons arise from neuroepithelial cells of the dorsal telencephalon, which confer glutamatergic over GABAergic transmitter identity through the sequential induction of *Pax6, Neurog1/2*, and *NeuroD* expressions (Fode et al., 2000; Gorski et al., 2002; Schuurmans et al., 2004; Kroll and O'Leary, 2005; Louvi et al., 2007). These cells then give rise to radial glial cells (RGCs), which possess characteristic apical and basal processes that make contact with the ventricular and pial surface, respectively. RGCs are the principal progenitor cells of the cerebral cortex (Malatesta et al., 2000; Miyata et al., 2001; Noctor et al., 2001) and also serve as scaffolds for the oriented migration of later-born neurons through their elongated processes. The progenitors contribute to cortical expansion in gyrencephalic mammals through the diversification of its subtypes (Hansen et al., 2010). RGCs undergo cell divisions at the ventricular surface that typically produce a pair of progenitors or a progenitor and a neuron. The former process is called symmetric cell division and expands the number of neural stem cells, whereas the latter is called asymmetric cell division and contributes to neurogenesis while maintaining the progenitor pool, owing to its output of both progenitor cells and neurons (and later glia). These progenitors are more fate-restricted in the sense that they have a limited capacity to undergo self-renewal.

The transition from neuroepithelial cells to RGCs is instructed through multiple signaling molecules. Fgf10, which is expressed in the apical surface of the VZ, exhibits a rostral-high to caudal-low gradient within the telencephalon, and genetic deletion of *Fgf10* results in delayed onset of RG markers, brain lipid binding protein (BLBP) and glutamate transporter (GLAST) in the rostral cortex. This delay results in the tangential expansion of prefrontal areas in the *Fgf10* mutants (Kang et al., 2009; Sahara and O'Leary, 2009), implying that the differential timing of neuroepithelial cell to RGC conversion may also contribute to the regulation of neuronal numbers in an area-dependent manner. Similarly, retinoic acid (RA) expressed in the meninges (Siegenthaler et al., 2009) instructs the conversion of division modes. Mutants that lack *Foxc1* fail to establish the meninges, which through contact with the end-feet of neuroepithelial cells propagate RA signaling, which is necessary for the transition from symmetric to asymmetric divisions. Lack of RA signaling derived from the meninges results in a significant decrease in neuronal output and thus prolonged neuroepithelial cell stage and symmetric cell divisions (Siegenthaler et al., 2009). Recently, a single-cell clonal analysis in mouse neocortex using retroviral vectors has demonstrated that, while the timing of transitions from symmetric to asymmetric cell divisions varies from clone to clone, within each clone, once the progenitors enter the asymmetric division phase, their progenies produce a remarkably fixed number of neurons (Gao et al., 2014). These observations revealed that following the conversion to asymmetric cell division mode, progenitor cells may undergo a stereotypic program in their proliferation and neurogenic output.

### Cajal-retzius cells and subplate cells in cortical scaffolding

When RGCs switch to asymmetric cell division, progenitor cells begin to produce the first cohort of neurons, which serve as essential scaffolds for the construction of the neocortical cytoarchitecture. These neurons consist of Cajal-Retzius (CR) cells and subplate (SP) neurons and form a transient structure called the preplate (PPL) above the VZ. CR cells were first recognized through their expression of secretory glycoprotein, Reelin (Reln) (D'Arcangelo et al., 1995; Ogawa et al., 1995), and the functional study of CR cells has largely focused on their regulation of radial migration in subsequent-born projection neurons through diffusive cues. However, recent reports have also revealed their roles in instructing radial migration via cell contact-mediated signaling (Gil-Sanz et al., 2013). Heterophilic cell adhesions mediated by nectin1-expressing CR cells stabilize the leading processes of nectin3-expressing migrating projection neurons to anchor to the MZ, facilitating their somal translocations toward the cortical surface. CR cells extend long horizontal axons within the MZ and also act as surface docking sites of synaptic contacts with branches of apical dendrites (Marin-Padilla, 1998; Meyer et al., 1999; Soriano and Del Rio, 2005). Recent reports have revealed the roles of CR cells in areal patterning and neurogenesis (Griveau et al., 2010; Teissier et al., 2012), indicating that CR cells have multimodal roles in instructing the early steps of cortical assembly.

In turn, the roles of SP cells in neocortical scaffolds were first revealed through ablation studies, in which SP cells in cats were eliminated using kainate. These experiments demonstrated that lateral geniculate neuron (LGN) axons fail to innervate their normal targets, which are layer 4 thalamorecipient neurons in the visual cortex (Ghosh et al., 1990). An interesting experiment to shift the tangential alignment of SP and overlaying primary somatosensory area (S1) layer 4 neurons through the electroporation of Fgf8 in the E11.5 mouse neocortex has revealed that thalamocortical axons can still recognize and innervate layer 4 cells via contact with SP neurons, albeit in a positionally shifted manner (Shimogori and Grove, 2005). Together with the observation that thalamic axons relay through superficially mispositioned SP cells in the *reeler* mutants (Molnar et al., 1998), these results indicate the primary roles of SP cells in guiding thalamic axons to enter the cortical plate (CP) and respond to cues provided by layer 4 neurons. SP cells also act as a gateway for neurons to enter the overlaying CP and accommodate massive numbers of neurons during and after their migration, thereby serving as a physical border between the CP and the intermediate zone (IZ). Perturbations in the expression of multiple genes in postmitotic cells result in halted migration and accumulation of neurons in the IZ (Miyoshi and Fishell, 2012; Ohtaka-Maruyama et al., 2013). SP cells are also required to assemble the functional neocortical circuit, where the ablation of SP cells disrupts the formation of ocular dominance columns (Ghosh and Shatz, 1992; Kanold et al., 2003). Although the molecular functions of SP cells have yet to be identified, extensive transcriptome analysis has revealed multiple cell surface components and secretory molecules that are expressed in both mouse and human SP cells, including CTGF, Cdh18, Efna5 (Mackarehtschian et al., 1999; Oeschger et al., 2012; Hoerder-Suabedissen and Molnar, 2013; Miller et al., 2014).

These tangentially coordinated CR cells and SP cells, with vertically oriented RGC fibers, form a perpendicular meshwork that enables the efficient weaving (integration) of newly generated layers of 6 to 2/3 neurons above their recently diverged siblings. In this view, the longitudinal radial glia serve as the warp and horizontally piled layer neurons serve as the weft to enable compacted neuronal accumulation and stratified CP. This process facilitates the efficient compression of massive number of neurons within a hard-boned skull-constrained space. RGCs, CR cells, and SP cells are also characteristic cell types of mammalian vertebrates, indicating that the appearance of these scaffolds instructed a neocortex-type laminated brain structure specifically in mammals. The numbers of CR cells and SP cells also expand during the course of mammalian evolution, suggesting that these neurons may have contributed to robust intercortical connectivity in primates (Smart et al., 2002; Molnar et al., 2006; Cabrera-Socorro et al., 2007). While many of these scaffolding cells are eliminated during the early postnatal period (del Rio et al., 1995; Price et al., 1997; Soda et al., 2003), a proportion of CR cells and SP cells survive in the postnatal neocortex, suggesting that these neurons also play roles in modulating the mature neocortical circuit (Kostovic and Rakic, 1980; Chowdhury et al., 2010; Judas et al., 2010).

## Molecular mechanisms of neuronal identity transitions

Following the dispositions of the preplate cells and conversion from symmetric to asymmetric cell division, RGCs begin to produce layer projection neurons through sequential rounds of cell cycles (Takahashi et al., 1999). Neurons are successively generated and migrate past the pre-existing neurons to occupy the more superficial layers, resulting in an inside-out lamination of the neocortex (Angevine and Sidman, 1961). Therefore, neuronal birthdate is highly correlated with final laminar fate, in which neurons that occupy the same radial positions are typically generated within the same temporal window and share common projection targets. Deep-layer (DL) neurons, which include layers 5 and 6, consist mainly of corticofugal projection neurons and project to subcortical targets. These neurons express transcription factors Fezf2, Ctip2, Tbr1, or Sox5 (Hevner et al., 2001; Arlotta et al., 2005; Kwan et al., 2008; Lai et al., 2008; Han et al., 2011; McKenna et al., 2011), according to their projection subtypes, including the spinal cord, tectum, and thalamus (Hirata et al., 2004; Inoue et al., 2004; Chen et al., 2005a,b, 2008; Molyneaux et al., 2005; Molnar and Cheung, 2006; Yoneshima et al., 2006). In turn, upper-layer (UL) neurons, which include layer 2/3 projection neurons and layer 4 thalamorecipient neurons process higher-order information through intracortical connections. Layer 2/3 neurons typically express the transcription factors Cux1/2, Brn1/2, Satb2 (McEvilly et al., 2002; Sugitani et al., 2002; Nieto et al., 2004; Alcamo et al., 2008; Britanova et al., 2008; Franco et al., 2012) and project their axons to the ipsilateral and contralateral cortex, thereby establishing bilateral cortical connections and information integration. Layer 4 neurons, in turn, are recipient cells for thalamocortical inputs and act as a gateway for processing information from peripheral sensory organs. Layer 4 neurons typically exhibit unique cellular arrangements in the primary sensory areas, maintaining topographic organization mediated through sensory transfer. Here, we focus exclusively on understanding the mechanisms that regulate the specification and transitions between the major layer subtypes of the neocortex.

### Cell competence and lineage restrictions

**Figure 1:**
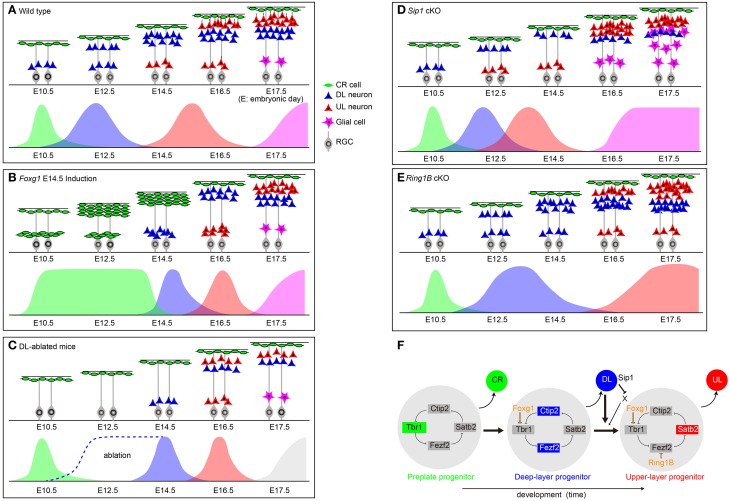
**Mouse mutants that exhibit shifts in temporal subtype transitions**. **(A–E)** Boxes indicate temporal progression of neurogenesis in wildtype and conditional knockout mice and in DL-ablated mutant mice. Bottom scheme in each box indicates production of respective subtypes based on representative birthdating experiments depicted from each mutant analysis. **(B)** Foxg1 E14.5 induction: analysis from E9.5 to 14.5 Foxg1 OFF in *Foxg1*^*tetOFoxg*1^ mice (Kumamoto et al., 2013; Toma et al., 2014), **(C)** DL-ablated mice: mice in which newly-born DL neurons were ablated through consecutive tamoxifen administration at E11.5, E12.5, E13.5 in *Neurog2*^*CreER*∕+^; *Rosa*-stop*-DTA* mice (Toma et al., 2014). These mutants have not been assessed for glial production. **(D)** *Sip1* cKO: analysis from *Nestin-Cre*; *Sip1*^*flox*∕*flox*^ or *NEX-Cre*; *Sip1*^*flox*∕*flox*^ conditional knockout mice (Seuntjens et al., 2009), **(E)** Ring1B cKO mice: analysis from *NestinCreERT2; Ring1B*^*flox*∕*flox*^ mice administered tamoxifen at E13.0 (Morimoto-Suzki et al., 2014) and E13.5 (Hirabayashi et al., 2009). CR, Cajal-Retzius; DL, deep-layer; UL, upper-layer; RGC, radial glial cell; cKO, conditional knockout. **(F)** Molecular mechanisms of neuronal subtype transitions during corticogenesis. Cortical progenitor cells at earliest stage express multiple transcription factors including Tbr1 and differentiate to CR cells. Induction of Foxg1 by FGF8 represses Tbr1 in the layer transcriptional network, switching the progenitor fate to DL production. The transition from DL to UL neurons is regulated by signals propagated from postmitotic DL neurons, terminating DL production through negative feedback. However, DL neurons also express Sip1, which represses DL to UL transition through presumptive downstream molecule(s) X, in which the progressive accumulation of these molecule(s) may facilitate DL to UL and subsequent UL to gliogenesis transitions. The H3K27me3 level and Ring1B binding at the *Fezf2* promoter also increases over time, facilitating the DL-UL transition.

The earliest assessment of temporal neurogenesis in the cerebral cortex was achieved through birthdating studies using tritiated thymidine injection in mice and monkeys. These experiments revealed that neocortical layer neurons are produced in a fixed temporal order (Angevine and Sidman, 1961; Rakic, 1974), implying that once progenitors switch to asymmetric cell division mode, they undergo progressive changes in competence to generate distinct layer subtypes (Figure 1A) (Takahashi et al., 1999). This strictly ordered production has raised several hypotheses concerning the mechanisms by which distinct layer subtypes arise from a small number of progenitor cells. McConnell and colleagues were the first to experimentally test the temporal differentiation capacity of cortical progenitors, using a series of isochronic and heterochronic cell transplantation in ferret cortices. The major findings from these studies were that, while early-born DL progenitors can adopt later (UL) cell fates upon transplantation to an older host environment, the converse manipulation could not induce later-born UL progenitors to adopt an earlier (DL) fate (McConnell, 1988; McConnell and Kaznowski, 1991; Frantz and McConnell, 1996). While subtype-specific markers were unavailable at the time, these studies were the first to demonstrate that the differentiation potency of progenitor cells is progressively restricted throughout the course of corticogenesis.

Aside from these transplantation experiments, examining the segregation mechanisms between laminar-specific subtypes involved complementary approaches to test their lineage relationships. Hence, extensive clonal analyses in mouse and rat cortex were performed to assess when and how the layer subtypes diverge during development. These studies revealed that at least a portion of progenitor cells, if not the majority, contribute to generating clones that encompass neurons of both deep and upper cortical layers (Luskin et al., 1988; Price and Thurlow, 1988; Walsh and Cepko, 1988, 1992; Reid et al., 1995; Yu et al., 2009; Gao et al., 2014). Furthermore, cell culture models testing the differentiation capacity of cortical progenitor cells *in vitro* also provided the basis for intrinsic and extrinsic mechanisms involved in these subtype transitions. *In vitro*, cortical cells also followed the general trend observed *in vivo*: DL neurons were commonly generated after fewer cell divisions than UL neurons in isolated cortical progenitors, and progenitors from later-stage embryos were more restricted in their ability to generate earlier-born neuronal subtypes (Shen et al., 2006). Furthermore, both mouse and human embryonic stem cell (ESC)- and induced pluripotent stem cell (iPSC)-derived cortical progenitors recapitulated the sequential generation of principal layer subtypes: preplate, DL, and UL neurons (Eiraku et al., 2008; Gaspard et al., 2008; Shi et al., 2012). These studies implied that the defined temporal order of projection neuron subtypes in the neocortex is controlled by temporal cues provided within the cortical cells themselves. Here, we discuss the identity of such cues that regulate the transitions between the major layer subtypes.

### CR cells to deep-layer neurons

Both *in vivo* and *in vitro*, the appearance of preplate neurons precedes the appearance of all other layer subtypes (Hevner et al., 2003; Eiraku et al., 2008; Gaspard et al., 2008; Shi et al., 2012). Here, preplate neurons are mainly CR cells based on the pan-CR cell marker Reln; thus far, no common marker for SP cells has been identified to test their differentiation capacity *in vitro*. Because of their earliest differentiation, a simple explanation concerning the ontogeny of CR cells may be that CR cell progenitors represent the default state of all cortical progenitors, thereby requiring minimum cues for their induction. However, several reports are discordant with this view: fate-mapping studies demonstrated that CR cells arise from discrete spatial domains, including the cortical hem, ventral pallium, thalamic eminence and septum, and these spatially distinct CR subtypes exhibit different molecular expressions (Bielle et al., 2005; Yoshida et al., 2006; Teissier et al., 2010; Zimmer et al., 2010). These observations implied that CR cells themselves already consist of different subtypes upon their differentiation. This discrepancy was later resolved through independent studies that assessed the temporal and spatial competence of CR cells, revealing that the distinct CR origins were commonly repressed by transcription factors Foxg1 (Kumamoto et al., 2013) and Lhx2 (Roy et al., 2014). Through a series of gene knockout studies, the removal of either of Foxg1 and Lhx2 at developmental onset resulted in the expansion of CR origins of cortical hem-, ventral pallium- and thalamic eminence-derived character (Hanashima et al., 2007; Mangale et al., 2008; Kumamoto et al., 2013; Roy et al., 2014). Interestingly, these transcription factors appear to act largely independently of each other, where their temporal knockout studies revealed an earlier competence window of neocortical progenitors to revert to CR regional identities upon the loss of Lhx2 (E10.5–E11.5) compared to the loss of Foxg1 (E13) (Hanashima et al., 2007; Mangale et al., 2008; Chou et al., 2009; Kumamoto et al., 2013; Roy et al., 2014). These results were consistent with the distinct consensus binding sequences of these two transcription factors (Hatini et al., 1994; Wilson et al., 2008).

The termination of early CR cell production is instructed through combinatorial repression by Foxg1 and Lhx2; however, the mechanisms by which progenitor cells switch from CR cell to DL neuron production required further mechanistic insights. Although the primary targets of Lhx2 involved in this event remain to be identified, the transcriptional regulatory network underlying this early subtype transition was revealed through an experiment in which Foxg1 expression onset was synchronously manipulated in cortical progenitors *in vivo*. When Foxg1 was induced at a progressively later stage during the corticogenesis period, progenitors converted to producing DL neurons (Kumamoto et al., 2013), enabling the examination of the temporal gene expression dynamics within the progenitors involved in this transition. These genome-wide studies revealed that the switch from CR cells to DL neurons involves the rapid repression of multiple transcription factors, followed by the delayed induction of upregulated transcription factors (Kumamoto et al., 2013). These results also demonstrated that the progenitor cells of CR cell and DL neuron fates share a common competence window, in which Foxg1 is both necessary and sufficient to confer the DL neuron fate over the CR cell fate. Taken together, the earliest transition of CR-to-DL neurons requires two sequential steps, which are mediated through the suppression of CR cell identity and the switch to projection neuron fate through the Foxg1 downstream cascade followed by cross-regulatory determination within layer neurons through subtype-specific determinants. Foxg1 itself is induced by FGF8 expressed in the anterior neural ridge (Shimamura and Rubenstein, 1997) and subsequently expands caudally, thus the onset of Foxg1 expression represses multiple transcription factors in an opposing rostral-to-caudal gradient, resulting in a spatiotemporal switch from CR cell to DL neuron identity (Kumamoto et al., 2013). This process also implies that the expansion timing of Foxg1 determines the total number of CR cells produced in the cortex, which provides a mechanism to generate sufficient numbers of CR cells to cover the entire surface area prior to the onset of DL neurogenesis and to instruct the migration of later-born projection neurons.

### Deep-layer to upper-layer neurons

In contrast to the transition from CR cells to DL neurons, which is mediated by Foxg1 and its downstream gene network, the switch from DL to UL neurons appears to utilize multiple regulatory cascades. In the aforementioned *Foxg1* conditional mutant mice, the induction of Foxg1 at progressively later stages during development (E14.5–E16.5) showed that UL progenitors are unable to bypass DL competence for their production even at the latest period of corticogenesis (Toma et al., 2014) (Figure 1B). The emergence of UL neurons was also assessed through lineage studies, in which Foxg1 and Cre constructs were introduced into *Foxg1*^−∕−^*; Rosa26*-stop-*YFP* mice, thereby labeling all progeny of Foxg1-introduced progenitors. These studies revealed that both DL and UL neurons were labeled with YFP, which determined that UL neurons emerge from cells with a Foxg1-lineage after the onset of Foxg1 expression (Toma et al., 2014). Birth-dating studies further confirmed that UL generation followed DL neurogenesis in these cells, demonstrating that the cascade downstream of Foxg1 triggers the sequence of DL and UL neuron production. These results also indicated that neocortical progenitors were biased toward DL over UL neuron fate upon Foxg1 induction.

The molecular logic underlying this DL neuron fate bias of progenitors was again uncovered through Foxg1 downstream transcriptome analysis. Of the layer transcription factors, Tbr1, which is expressed in the majority of early-born neurons (Hevner et al., 2001) and establish the corticothalamic projection neuron identity within the layer-subtype transcriptional network (Han et al., 2011; McKenna et al., 2011; Srinivasan et al., 2012), exhibited a significant downregulated response to Foxg1 induction. A reporter assay revealed that this repression was mediated through a 4-kb Tbr1 promoter region consisting of multiple conserved Foxg1 binding sequences. The introduction of *Foxg1* into E14.5 *Foxg1*^−∕−^ cortices demonstrated that this downregulation of Tbr1 preceded the onset of Ctip2 and Fezf2 protein induction (Toma et al., 2014). Collectively, these data show that Tbr1 repression by Foxg1 confers the sequence of DL and UL competence by establishing the bias to DL (Fezf2^ON^/Satb2^OFF^/Ctip2^HI^) identity (blue cells indicated in Figure 1F).

The subsequent transition from DL to UL neurogenesis requires the repression of DL determinants to terminate DL competence, which involves both negative feedback and epigenetic regulations. In this regard, in experiments with the ablation of post-mitotic DL neurons *in vivo*, the relative DL neuron:UL neuron ratio was maintained despite the ablation of a significant number of DL neurons. The injection of EdU to monitor the neurons that were born from progenitors in post-ablated cortices revealed that the ablation of DL neurons prolonged the production period of DL neurons themselves, and UL neurons born at E14.5 also decreased alongside increased DL production (Figure 1C). Collectively, these results demonstrate that the onset of UL neuron generation is controlled by the termination of DL competence, which is propagated through post-mitotic DL neurons (Toma et al., 2014). Interestingly, this signal appears to act qualitatively rather than quantitatively *in vivo*, where only a few postmitotic DL neurons are required to induce UL neurogenesis (Toma et al., 2014), in contrast to the requirements *in vitro* (Shen et al., 2006; Eiraku et al., 2008; Gaspard et al., 2008; Kadoshima et al., 2013). These observations raise the possibility that this feedback signaling may be propagated by short-range signaling through cell–cell interactions.

While these studies showed that both DL and UL lineages are generated downstream of the Foxg1 cascade, whether the generation timing differences between the DL and UL neurons are achieved through temporal changes in competence within common progenitors (Guo et al., 2013; Gao et al., 2014; Eckler et al., 2015) or through extended mitosis specifically in early UL-committed cells (Franco et al., 2012; Gil-Sanz et al., 2015) is unclear. Because the termination of DL competence is required for both cases, negative feedback from postmitotic neurons appears to be the primary source of this cue, whereas in the latter model, additional mechanisms are required to extend mitosis in UL-committed cells. Although the decrease in UL neurons generated with extended DL neurogenesis upon ablation of DL neurons suggests the presence of common progenitors that can contribute to both DL and UL neurons, it is possible that the prolonged DL production in DL-ablated cortices may result in extended proliferative cues for UL cells. This regulation has been suggested in Sip1-expressing postmitotic neurons that maintain low expression levels of multiple secretory protein genes, including Ntf3 (Seuntjens et al., 2009; Parthasarathy et al., 2014). The accumulation of these proteins may be required to induce the differentiation of UL progenitors (Seuntjens et al., 2009) (Figure 1D). In this case, since *Ntf3* knockout alone or *Ntf3; Sip1* double knockout mice do not exhibit changes in the Ctip2^+^ neuron:Satb2^+^ neuron ratio compared with wild-type or *Sip1* knockouts, respectively (Parthasarathy et al., 2014), Sip1 may act through the repression of additional molecule(s) in this event (factor X indicated in Figure 1F). The extended DL neurogenesis achieved through the ablation of DL neurons may itself sustain low levels of these signaling molecules, thereby maintaining the UL-committed cells as progenitor cells for a prolonged period of time. As UL projection neurons mediate higher-order information processing, and their numbers expand in gyrencephalic mammals (Aboitiz and Montiel, 2003; Schoenemann et al., 2005), these feedback mechanisms also provide a new perspective as to how cell type transitions adapt to increases in cortical size, gestational period, cell cycle, and division modes (Fietz and Huttner, 2011; Lui et al., 2011) to balance the production of UL with DL neurons in different mammalian species (Striedter, 2005; Abdel-Mannan et al., 2008).

Studies have indicated that the transition from DL to UL neurogenesis is also controlled by epigenetic mechanisms. Ring1B, a component of the polycomb-repressing complex, represses Fezf2 expression in the late corticogenesis phase to shift the progenitor competence from DL to UL neurons. In knockouts that disrupt the expression of Ring1B, Ctip2^+^ DL neurons are increased and Cux1^+^ UL neurons are decreased (Morimoto-Suzki et al., 2014) (Figure 1E). During this process, the H3K27me3 epigenetic mark is increased on the promoter region of Fezf2, and Ring1B binds to this marked region to suppress *Fezf2* gene expression (Figure 1F). In turn, in mutants in which ESET histone methyltransferase was ablated, the population of UL neurons expands at the expense of DL neurons (Tan et al., 2012). This accelerated UL production, however, prematurely decelerates at E16.5, which is the peak of normal UL neurogenesis. As a result, the production of UL neuron numbers is not significantly affected. Because neuronal survival and proliferation is also affected in ESET cKO mice, ESET may regulate the transition from DL to UL neurons indirectly through these events (Tan et al., 2012). In the future, studies that examine gene locus-specific and time-dependent mechanisms that regulate chromatin modification will likely provide further insights into the epigenetic mechanisms that govern temporal neuronal identity transitions.

### Upper-layer neurons to gliogenesis

The switch from UL neurons to gliogenesis represents the latest transition in corticogenesis; as this step involves the termination of neurogenesis, the timing of its transition determines the overall number of neurons produced in the neocortex. Here, we mainly refer to the transition from UL neurons to astrocytes, which are generated earlier than their glial counterparts, oligodendrocytes (Bayer and Altman, 1991; Jacobson, 1991). Dissociated cells from embryonic rodent brains revealed highly reproducible timing of the appearance of neurons and glia *in vitro*, and the generation of glia required fewer rounds of cell division in older cortex-derived progenitors than in progenitors from younger cortex (Abney et al., 1981; Qian et al., 2000), demonstrating that this neuron-glia sequence was also preserved outside the cortical environment. The timing of the appearance of gliogenic clones and the relative proportions of neurons and glia that arise from a single cortical progenitor were also assessed through *in vivo* clonal analysis using retroviral vectors (Reid et al., 1995; Mione et al., 1997; Costa et al., 2009; Gao et al., 2014) and transgenic mice (Magavi et al., 2012). These studies indicated that both neuron-restricted and bipotent (that produce neurons and glia) progenitor cells appear early in the developing cortex (E10–E13 in mice) (Costa et al., 2009; Gao et al., 2014). Of all these labeled clones, approximately 16% were bipotent (Gao et al., 2014), implying that 1 out of 6 asymmetrically dividing clones proceed to gliogenesis after neurogenesis. In turn, glia-restricted progenitors were observed mainly in later stages of corticogenesis (Costa et al., 2009).

The sequential appearance of neurons and glia in isolated cortical cells has suggested several possible mechanisms underlying the transition from neurogenesis to gliogenesis. In particular, the behavior of these cells outside the cortical environment has demonstrated that temporal cues provided in culture were sufficient to drive these transitions. In this regard, key molecular pathways that direct progenitors toward neurons or astrocyte fate have been identified. Basic helix-loop-helix (bHLH) genes play redundant roles in repressing astrocyte identity during early- to mid-stage corticogenesis, where compound knockout of *Neurog2* and *Mash1* shows precocious astrocyte production at the expense of neurons (Nieto et al., 2001), and exogenous Neurog1 can increase the number of neurons and repress astrocyte differentiation (Sun et al., 2001). In turn, the differentiation of astrocytes is mainly activated through the Janus kinase-signal transducer and activator of transcription 3 (JAK-STAT3) pathway (Bonni et al., 1997). However, both JAK-STAT signaling components and activation ligands are present even during the neurogenesis phase (Molne et al., 2000), implying that the temporal switch from repression to activation of this pathway is crucial for the UL neuron to glia transition.

In this regard, polycomb group (PcG) protein-mediated epigenetic mechanisms play key roles in this transition. PcG proteins, which repress the *Neurog1* promoter in a developmental stage-dependent manner, suppress the *Neurog1* locus to restrict the neuronal competence of progenitors and promote the transition from neurogenesis to gliogenesis (Hirabayashi et al., 2009). The inactivation of PcG by knocking out *Ring1B* and *Ezh2* genes extends the neurogenesis period and delays the transition to astrocyte genesis (Hirabayashi et al., 2009). Interestingly, this shift in neuron-to-glia transition appears to depend on the time window of *Ezh2* removal: whereas conditional knockout of *Ezh2* at E12.5 results in a prolonged neurogenesis and delayed gliogenesis (Hirabayashi et al., 2009), the removal of *Ezh2* before the onset of neurogenesis results in the accelerated neurogenesis and also early onset of gliogenesis (Pereira et al., 2010). Thus, Ezh2 may independently regulate the switch from symmetric to asymmetric cell divisions in RGCs, which later alters the timing of neuron-to-glia switch in cortical progenitors. STAT signaling increases during the later corticogenesis phase through a positive autoregulatory feedback mechanism, thereby facilitating astrocyte production during the perinatal stages. The repression of astrocyte-specific genes during the neurogenesis period is also mediated through DNA methylation, in which DNA methyltransferase gene *DNMT1* knockout results in the upregulation of JAK–STAT signaling and early transition to astrocyte differentiation. Interestingly, the progenitor potential to switch from neurogenesis to gliogenesis is also regulated through a progressive global condensation of chromatin. The overexpression of the high-mobility group A proteins HMGA1 and HMGA2 in the E15.5 mouse neocortex maintains progenitors that express Tbr2, a marker for immature neuronal precursors, at a significantly late stage of corticogenesis (Kishi et al., 2012).

The latest transition from neurogenesis to gliogenesis also requires feedback mechanisms that instruct progenitors to switch competence from neurogenic to gliogenic progenitors. It has been reported that Fgf9, which is upregulated in postmitotic neurons during the later phase of the corticogenesis period, enhances the switch to gliogenic competence. In this regard, Sip1, which suppresses the expression of Fgf9 during the neurogenic period, is gradually downregulated during the progression of corticogenesis, which derepresses Fgf9 expression and facilitates the gliogenic competence transition (Seuntjens et al., 2009). Cardiotrophin-1 (CT-1), a member of the interleukin-6 family of neurotrophic cytokines, is also expressed in post-mitotic neurons and instructs the cortical progenitors to generate astrocytes through the gp130-JAK-STAT pathway. The introduction of this neurotrophic cytokine induces premature gliogenesis, whereas perturbations in the gp130-JAK-STAT pathway delay the onset of gliogenesis (Barnabé-Heider et al., 2005). Collectively, the transition from neurogenesis to gliogenesis utilizes compound regulatory cascades to progressively restrict the neurogenic potential of progenitor cells during the late stage of corticogenesis.

## Subtype-specific integration and neocortical assembly

Following the generation of diverse cell types, the precise integration of these cells is essential to the formation of the neocortical circuit. The migration of diverse neurons to a location away from their place of origin enables efficient wiring between distinct classes of neurons and promotes connection between the subtypes along the radial and tangential axis. Following the exit from the cell cycle, many neocortical neurons migrate along a stereotypic route from their place of origin to their final allocation; however, growing evidence has shown that distinct subtypes dynamically change patterns of migration en route by switching their responsiveness to temporal and spatial guidance cues. Here, we highlight such features that involve subtype-specific modes of neuronal integration during the assembly of the neocortex.

### Migration modes of preplate neurons

The patterns of neuronal migration of early-born preplate neurons have begun to be rapidly uncovered over the past years, illuminating their various integration routes upon entering the neocortical primordium. These disparate features likely reflect their molecular diversity acquired through their distinct spatial origins (Meyer et al., 1999, 2002; Griveau et al., 2010; Pedraza et al., 2014). As preplate neurons are the earliest neurons to migrate into the neocortex, they have the flexibility to move without much physical restriction in the absence of abundant radial glia or axonal fibers. While the spread of these neurons is clearly distinct from radial migration of later-born projection neurons, it is also somewhat different from a directional tangential migration, in which neurons exhibit a coordinated migration along the defined route, as observed in GABAergic interneurons from the ganglionic eminence to the cerebral cortex. The experimental evidence on the migration patterns of CR cells came first from fate-mapping studies of these neurons by the *exo utero* electroporation of lacZ-expressing plasmids in distinct regions of the pallium, where cells labeled in the dorsomedial pallium with lacZ migrate over the cortical surface through tangential dispersion (Takiguchi-Hayashi et al., 2004). These features were further confirmed by the genetic fate-mapping of distinct CR subtypes cells using *Wnt3a* and *Dbx1* knock-in mice (Bielle et al., 2005; Yoshida et al., 2006). These studies revealed that in addition to CR cells of the cortical hem (medial pallium) origin, the ventral pallium-derived CR cells also migrate along the surface of the developing cortex (Bielle et al., 2005). This behavior suggests that the “tangential spreading” property may be a fundamental feature of most CR cells generated from distinct sources.

**Figure 2:**
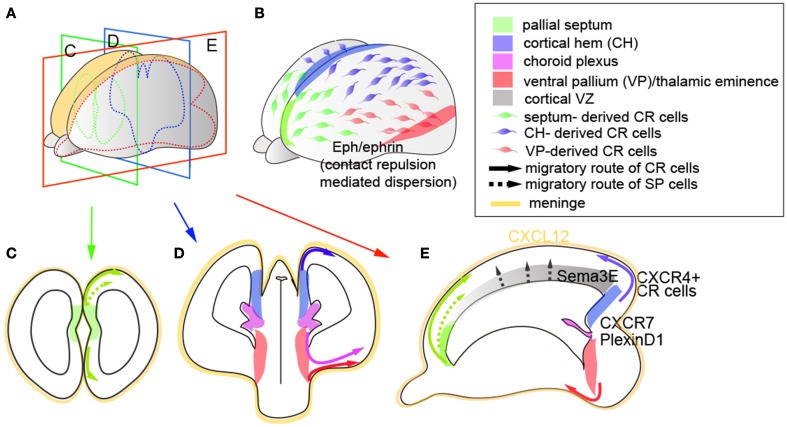
**Birth and integration of early-born preplate neurons**. **(A,B)** Whole view of the mouse neocortex. **(C,D)** Indicate coronal sections of **(A)** at rostral **(C)** and caudal **(D)** levels, **(E)** indicates tangential sections of **(A)**. Colored regions indicate respective domains of CR cell origins (pallial septum, cortical hem, choroid plexus, ventral pallium/thalamic eminence) and SP cell origins (pallial septum and cortical VZ). Lines and their colors indicate the migration routes of CR cells (solid lines) and SP cells (dashed lines) arising from respective regions. Yellow regions indicate meninges, which are the primary source of CXCL12 ligands. CXCR7 and PlexinD1 are expressed in most CR cells, whereas CXCR4 is predominantly expressed in cortical hem-derived CR cells. In addition, CR cells express multiple ephrin ligands and receptors, which act as contact-dependent repulsive cues within both homotypic and heterotypic CR cell subtypes. Sema3E is expressed in a caudomedial-high to rostrolateral-low gradient in the cortical VZ, which controls the pace of migration of CR cells that express PlexinD1.

Although CR cells arise from a relatively small district at the pallial border (Figures 2A,C,D), the unique surface spreading feature that enables CR cells to cover the entire cortical surface implies that the dispersion of these neurons may be achieved through either self-repulsive behavior and/or attractive cues provided along the route of their migration. Indeed, studies indicate that CR cells utilize both repellent and attractive cues to facilitate their dispersion along the tangential axis (Figures 2B,E). Here, both whole-mount cortical culture and mathematical modeling indicate that contact-mediated repulsion is necessary to optimize the cortical coverage of CR cells (Villar-Cerviño et al., 2013). In this study, CR cells of homotypic or heterotypic origins (i.e., cortical hem and ventral pallium or septum) (Figures 2C,D) exhibit similar repulsive responses, indicating that the CR cells of distinct sources can recognize each other to form spatial territories, mediated through the expression of multiple ephrin signaling molecules (Villar-Cerviño et al., 2013) (Figure 2B). This mechanism is consistent with the observation that CR cell coverage from distinct origins is highly compensatory, where ablation of either cortical hem-derived CR cells (Yoshida et al., 2006), septum-derived CR cells (Griveau et al., 2010), or combinatorial CR cell ablation of multiple sources (Tissir et al., 2009) results in the redistribution of alternative subtypes along the tangential axis. In these experiments, even upon the ablation of 84% of CR cells, Reln expression was still detectable at the cortical surface (Tissir et al., 2009), underpinning the highly compensatory features of Reln-expressing cells upon developmental perturbation. By contrast, the loss of septum-derived CR cells results in a shift in areal positioning during the postnatal stages (Griveau et al., 2010), suggesting that regional subtypes and their territorial disputes may be an important feature of neocortical tangential organization. In addition to these self-repulsive “tiling” properties, reports have indicated that CR cells also utilize attractive guidance cues. In particular, the chemokine CXCL12, expressed in the meninges, exerts its action through both of its receptors CXCR7 and CXCR4 to facilitate the surface migration of CR cells that express these receptors (Borrell and Marin, 2006; Trousse et al., 2014) (Figure 2E). The spatiotemporal expression of these receptors is slightly different: CXCR7 is expressed in most CR cells by E11.5 and later downregulated, whereas CXCR4 is predominantly expressed in cortical hem-derived CR cells at E11.5 and onward (Schönemeier et al., 2008; Tiveron et al., 2010), and knockout of either of these genes results in the ectopic distribution of a fraction of Reln-positive CR cells to deeper positions in the CP. Interestingly, CXCL12/CXCR4 signaling appears to be further modulated through Sema3E/PlexinD1 signaling, where the loss of PlexinD1 facilitates the migration of cortical hem-derived CR cells to more dorsomedial regions (Bribian et al., 2014) (Figure 2E).

In contrast to CR cells, the migration and integration properties of SP cells are worthy of further exploration. While the ontogeny of SP neurons has not been fully clarified, fate-mapping studies imply that these neurons contain at least two distinct lineages (Gao et al., 2014; Pedraza et al., 2014). Retroviral lineage tracing revealed a proportion of SP cells co-labeled with DL and UL neurons in the neocortex, indicating the common lineage between these subtypes and the cortical VZ origin of SP cells (Gao et al., 2014) (Figure 2E). However, SP cells have also been observed at the pallial boundary; specifically, a subpopulation of SP cells arises from the rostromedial pallium (Pedraza et al., 2014) and migrates dorsally to invade the cortex (Figure 2C). The diversity in their molecular repertoire and ontogeny (Miller et al., 2014) implies that SP cells may also possess subtype-specific integration and function during cortical assembly, and merits further study.

### Radial integration of neocortical subtypes

The lamination of the cerebral cortex is largely attributed to the unique radial migrating feature of projection neurons in the mammalian brain system, in which identical migration modes have not been observed thus far in other amniote cortices (Nomura et al., 2008, 2013b; Lui et al., 2011; Jarvis et al., 2013; Montiel and Molnar, 2013). This feature contributes to the distinctive cytoarchitecture of the neocortex and neural processing in mammalian vertebrates, despite the conserved components of neuronal subtypes based on gene expression and connectivity patterns (Suzuki et al., 2012; Jarvis et al., 2013; Nomura et al., 2013a).

**Figure 3:**
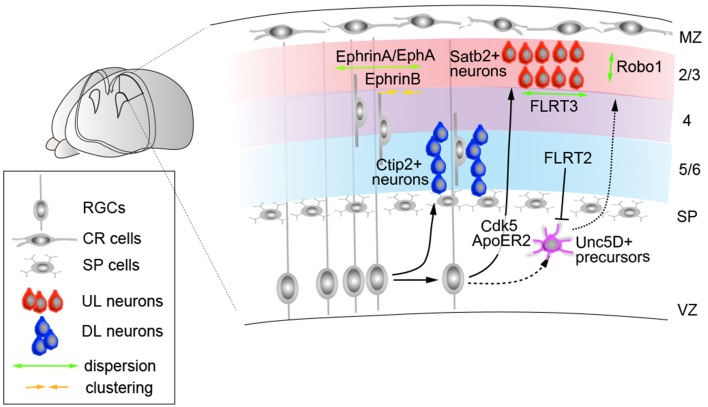
**Molecules that control subtype-dependent cortical neuron integration**. The migration and distribution along the radial and tangential axis are regulated by multiple ligand/receptor molecules expressed in neocortical subtypes. In deep layers, Ctip2-positive subcerebral projection neurons form periodic organizations in layer 5, observed in both the mouse and human neocortex. In humans, the expression segregation of NOS1 in these columns is regulated by FMRP. Upper-layer neurons require Cdk5 and ApoER2 for their migration. Within the upper-layers, Satb2-, and Unc5d-positive neurons represent distinct subpopulations. The latter exhibits delayed integration into the CP through repulsive interactions, with high FLRT2 expression at E15.5 that decreases perinatally. In turn, FLRT3 regulates the tangential dispersion of E15.5-born UL neurons through adhesive interactions. In turn, the radial distribution of Satb2-positive UL neurons is regulated by Robo1. Apart from these molecules, the tangential integration of neocortical neurons is regulated through multiple ephrin-As, which facilitate lateral dispersion of both DL and UL neurons. Ephrin-B1 reverse signaling, in turn, is required to limit the tangential dispersion of ontogenic columns derived from E13.5-born progenitor cells. MZ, marginal zone; SP, subplate.

In general, the patterns of birth and migration of cortical projection neurons are considered to conform the following rules: each layer of neurons arises from the VZ and SVZ progenitors and moves radially toward the pial surface via multi-step guided migration processes. Broadly, this process involves a series of migration and positioning events, including multipolar-to-bipolar transition (Tabata and Nakajima, 2003; Noctor et al., 2004; Tabata et al., 2009), radial glia-guided locomotion (Rakic, 1972; O'Rourke et al., 1992; Nadarajah et al., 2001), detachment from radial glia (Pinto-Lord, 1982; Gongidi et al., 2004; Elias et al., 2007), and terminal somal translocation (Nadarajah et al., 2001; Sekine et al., 2011). The repetition of these events by sequential cohorts of neurons enables newly born neurons to migrate past their predecessors and take a more superficial position within the CP, establishing an “inside-out” neuronal distribution pattern (Angevine and Sidman, 1961; Rakic, 1974). The earliest evidence that layer projection neurons may utilize a subtype-specific migration mode came from a time-lapse imaging study of mouse cortical slices obtained from different developmental stages (E13–16) and labeled with Oregon Green to visualize individual neurons (Nadarajah et al., 2001). These experiments revealed that early-born subtypes predominantly undergo somal translocation to move toward the pia, which is later replaced with radial glia-guided locomotion events. The switch in these events is correlated with the overall increase in distance from the ventricular zone to the pial surface, where early-generated DL neurons require a shorter distance to migrate using extended basal processes. Consistent with this view, DL and UL progenitors appear to use distinct molecular machineries to enter the CP, in which UL but not DL neurons are susceptible to the loss of cyclin-dependent kinase 5 (Cdk5) activity (Hatanaka et al., 2004) (Figure 3). Furthermore, while Reln is required for both DL and UL neuron migration, its signal propagation appears to be mediated through distinct receptors between these subtypes; apolipoprotein receptor 2 (ApoER2) knockout mice exhibit a defect in Cux2-positive UL neurons but not ER81-positive DL neurons (Hack et al., 2007). Consistent with this observation, a recent expression study has demonstrated that ApoER2 protein is predominantly upregulated in postmitotic cells during the UL neurogenesis period (Hirota et al., 2015) (Figure 3).

Studies have also suggested that the timing of the CP entry of cortical projection neurons may also be instructed through subtype-specific mechanisms. Expression and loss-of-function studies have indicated that the UL neurons of the neocortex include at least two subpopulations, Satb2^+^ and Unc5d^+^ neurons; whereas Satb2^+^ neurons migrate toward the CP immediately after their cell cycle exit, Unc5D-positive cells undergo a longer waiting period (3–4 days) within the SVZ (Tarabykin et al., 2001; Britanova et al., 2008) (Figure 3). The knockout mutant of both *Unc5D* and its interacting fibronectin and leucine-rich transmembrane protein-2 (*FLRT2*) exhibits the acceleration of these neurons to migrate toward the CP (Yamagishi et al., 2011), implying that the timing of integration of UL neurons is determined through subtype-dependent molecular cues.

Currently, increasing numbers of molecules have been identified that control the early phase of radial migration (Caviness and Rakic, 1978; Gupta et al., 2002; Nadarajah and Parnavelas, 2002; Tsai and Gleeson, 2005; Cooper, 2008; Huang, 2009; Honda et al., 2011); however, little is known about how the terminal positioning of neuronal subtypes is established after they arrive at the surface of the CP. The conditional ablation of genes encoding the alpha subunits of heteromeric G proteins G_12_ and G_13_ has shown that neurons cause overmigration at the cortical surface despite the intact organization of CR cells, RGCs, and basal lamina (Moers et al., 2008). In these mutants, the positioning defect appear only in a restricted number of neurons, suggesting that alternative mechanisms may also contribute to this event. In this context, Robo1, a member of the family of Roundabout receptors, regulates the radial dispersion of UL neurons in the neocortex (Figure 3). In a series of knockout and knockdown studies, the suppression of Robo1 was shown to result in E15-born neurons predominantly localizing to the uppermost part of layers 2/3, in contrast to control cells that were distributed radially in these layers. The sequential electroporation of fluorescent reporter constructs revealed that Robo1-suppressed cells fail to establish the characteristic inside-out neuronal distribution and accumulate beneath the marginal zone, also resulting in a thinner CP, as observed in *Robo1* knockouts. Temporal analysis also reveals that E14.5-born cells, unlike E15.5 or E16.5 neurons, do not exhibit changes in their positioning upon Robo1 suppression. As the majority of E14-born neurons adopt a layer 4 fate (Takahashi et al., 1999) and normally do not express detectable levels of Robo1 (Gonda et al., 2013), these results imply that Robo signaling acts in a subtype-restricted manner, where layer 4 neurons are refractory to loss of Robo1 expression. Collectively, these studies suggest that the mechanisms by which projection neurons migrate and integrate to their radial positions are regulated through subtype-specific codes that refine the formation of neocortical layers.

### Tangential dispersion of neocortical neurons

Following extensive histological studies of Golgi impregnated brains, the periodic neuronal arrangements within the cerebral cortex have motivated scientists to decipher the spatial and functional codes that drive the circuit of the neocortex. However, in contrast to the discernible laminar organization of neocortical neurons (Brodmann, 1909), the existence of definable anatomical cellular organization across tangential dimensions has remained less clear. Following Lorente de No's hypothesis of translaminar cellular modules, Mountcastle (1957) proposed that vertical columns of neurons in the cerebral cortex are fundamental processing units of the neocortex, a theory inherited by Hubel and Wiesel, leading the concept of cortical modules and receptive fields. Although electrical recordings have revealed functional clustering and neuronal interactions along the cortical tangential dimensions, whether such modules could be defined by their anatomical and molecular character has remained elusive. However, it is increasingly becoming clear that multiple molecules may contribute to the efficient tangential mixing of neocortical projection neurons.

The functional analysis of Ephrin signaling has demonstrated that Eph receptor A (EphA) and ephrin A (Efna) signaling are essential for the assembly of cortical columns through the lateral dispersion of clonally related neurons (Torii et al., 2009) (Figure 3). Furthermore, a recent study revealed that ephrin-B1 also regulates the tangential motility of projection neurons, where gain-of-function of ephrin-B1 results in abnormal neuronal clustering. Conversely, ephrin-B1 knockouts display a wider lateral dispersion, resulting in the enlargement of ontogenic columns (Dimidschstein et al., 2013) (Figure 3). Similarly, FLRT-mediated signaling has also been shown to regulate the early tangential spread of projection neurons, in which abnormal neuronal clustering of E15.5-born neurons was observed in the tangential but not the radial axis in *FLRT3* conditional knockout mice. Together, these observations established the molecular basis that facilitates the tangential arrangement of neocortical projection neurons in general (Figure 3).

In this context, several reports have also indicated subtype-specific mechanisms for tangential neuronal dispersions. DL projection neurons, particularly the subcerebral projection subtypes within layer 5, that express markers including CTIP2 and FEZF2 and nitric oxide synthase 1 (NOS1) are segregated in periodic arrangements across the tangential dimensions (Maruoka et al., 2011; Kwan et al., 2012) (Figure 3). In both the developing mouse and human cortex, these neurons also exhibit high expression correlation with the neuronal activity marker c-Fos (Maruoka et al., 2011; Kwan et al., 2012). In mice, these microcolumns appear to comprise multiple clones, in agreement with clonal studies indicating more radially dispersed neurons of sister neurons arising from a single progenitor origin (Yu et al., 2009). Interestingly, in humans, this periodic segregation of layer 5 gene expression appears to be instructed in an area-specific manner, through the translational regulation of NOS1 by RNA-binding protein FMRP. Whereas, NOS1 mRNA is ubiquitously expressed, NOS1 protein is transiently co-expressed with FMRP during the early synaptogenesis period in layer 5 neurons of the prospective Broca's area and orofacial motor cortex (Kwan et al., 2012). The translation of NOS1 is activated by FMRP via interactions with binding motifs that are absent in mouse *Nos1* mRNA, implying that while periodic arrangements are common features of mouse and human subcerebral projection neurons, subsets of their gene expressions may be regulated in a species- and area-dependent manner. These alterations to gene expression regulation in the developing neocortical circuit may also contribute to cognitive dysfunctions in X fragile syndrome caused by mutations in FMRP coding gene *FMR1* (Ashley et al., 1993).

Studies have demonstrated that Reln, in addition to their roles in instructing radial neuronal migration, also plays important roles in the tangential migration of layer projection neuron subtypes (Britanova et al., 2006). Migration assay using wildtype mouse brain slices revealed that Satb2^+^ projection neurons, derived from local neocortical progenitors, migrate tangentially within the upper IZ over long distances; however in *reeler* mice this migration was impaired, resulting in the reduced number of Satb2^+^ cells in the subiculum (Britanova et al., 2006). Because the tangential migration of interneurons is not affected in *reeler* mice (Hevner et al., 2004), Reln appears to be specifically required for the tangential migration of Satb2^+^ projection neuron subtypes. Furthermore, a recent study demonstrated that the disruption of *Reln* or its receptor *Dab1* expression, or overexpression of Ephrin-A signaling components, all disrupted the preferential electrical coupling between the radially aligned sister excitatory neurons, which are normally observed during development (Yu et al., 2009, 2012; He et al., 2015). Thus, the extent of tangential dispersion of newborn neurons within and across the cortical subtypes, may be a critical determinant for instructing the neuronal connectivity during the initial phase of cortical circuit assembly.

### Areal patterning of neocortical neurons

In addition to the segregation of the laminar subtypes, which is achieved through cross-repressive interactions between multiple transcription factors, it is becoming increasingly evident that transcription factors also play pivotal roles in establishing the regional identity of the neocortex, referred to as cortical arealization. Seminal work examining the function of transcription factors Emx2, Pax6, and Sp8, have revealed that the graded expression of these genes within cortical progenitors and their genetic interactions is required for establishing the topographic organization of neocortical areas (Bishop et al., 2000; Muzio et al., 2002; Hamasaki et al., 2004; Sahara et al., 2007; Zembrzycki et al., 2007, 2013). Notably, the regional characters acquired in cortical progenitors are susceptible to subsequent gene expression changes in post-mitotic neurons. Conditional knockout of *COUP-TFI*, an orphan nuclear receptor expressed in a caudal-high to rostral-low gradient in the developing forebrain (Qiu et al., 1994), results in the expansion of the frontal cortex at the expense of a compressed occipital cortex (Armentano et al., 2007). Interestingly, this caudal-to-rostral shift in cortical identity is also observed in mouse mutants in which *COUP-TFI* was specifically removed in post-mitotic neurons (Alfano et al., 2014). Conversely, the expression of *COUP-TFI* in post-mitotic neurons appears necessary and sufficient to restore the area-specific expression patterns of genes including *Cadherin-8, Bhlhb5*, and *Id2* (Alfano et al., 2014). Similarly, *Bhlhb5*, a bHLH gene expressed in a caudomedial-high to rostrolateral-low gradient in the post-mitotic neurons, is required to establish the regional expression of *COUP-TFI, RORb, Id2*, and *Cadherin-8* (Joshi et al., 2008). Therefore, COUP-TFI and Bhlhb5 are not only responsible for establishing areal patterning of neocortical neurons, but are also reciprocally required for their regional and laminar-specific gene expressions (Joshi et al., 2008; Alfano et al., 2014). Although the downstream mechanisms by which these transcription factors confer the area-specific neuronal distribution remain to be explored, these results suggest that cortical layer subtypes utilize region-specific cues to integrate into distinct cortical areas, which may contribute to different laminar thicknesses among neocortical areas.

## Perspectives

### Neurological disorders associated with cortical assembly defects

The increased number of genes identified in their functions for the generation and integration of neocortical subtypes, has provided molecular link between neurological disorders with corresponding gene mutations and mechanisms underlying pathogenesis. Apart from the aforementioned fragile X syndrome causative gene *FMR1*, perturbations of genes that play key roles in the differentiation of neocortical layer subtypes have been associated with a wide spectrum of neurological phenotypes. Screening for *de novo* mutations in patients with intellectual disability have identified Foxg1 and Tbr1, two of the transcriptional regulatory network components for layer subtype specification (see Section Deep-layer to Upper-layer Neurons and Figure 1F) as altered in their gene sequences (Hamdan et al., 2014). Loss-of-function variants (point mutations, deletions, and *de novo* translocations) and gene duplications of *FOXG1* have been associated with phenotypes including developmental epilepsy, agenesis of the corpus callosum, microcephaly, and speech impairment (Shoichet et al., 2005; Bisgaard et al., 2006; Papa et al., 2008; Yeung et al., 2009; Bahi-Buisson et al., 2010; Mencarelli et al., 2010; Brunetti-Pierri et al., 2011). In turn, its repression target *TBR1* has also been identified as one of the genes with recurrent *de novo* mutations in autism spectrum disorders (ASD) (O'Roak et al., 2012). Coexpression network analysis to identify the time period and regional convergence of high-confidence ASD genes, revealed *TBR1* as the most connected ASD gene within the key convergence point in human midfetal layers 5/6 projection neurons (Willsey et al., 2013). The functional implications of the identified *de novo* mutations, were assessed by introducing the corresponding *TBR1* gene mutations into HEK293 and SHSY5Y cell lines (Deriziotis et al., 2014). These experiments resulted in the disruption of subcellular localization of TBR1 and interaction with CASK, a membrane-associated guanylate kinase also involved in ASD (Moog et al., 2011). Similarly *SATB2*, an evolutionary conserved chromatin remodeling gene that is activated in UL neurogenesis and required for callosal projection subtype determination (Section Deep-layer to Upper-layer Neurons and Figure 1F), is a key gene for the 2q33.1 microdeletion syndrome (Rosenfeld et al., 2009), and *SATB2* haploinsufficiency has been associated with significant speech delay and cognitive defects (FitzPatrick et al., 2003; Leoyklang et al., 2007; Usui et al., 2013; Döcker et al., 2014).

Taken together, subtle mutations in the corresponding genes can result in profound neurodevelopmental disorders in humans; however, studies in mouse neocortex have also revealed a high compensatory feature of neurogenesis upon robust ablation of its subpopulations. Up to 84% of CR cell ablation does not demolish Reln expression in the neocortex (Tissir et al., 2009), and ablation of a significant number of DL neurons still preserves the DL:UL neuron ratio at later stages of corticogenesis (Toma et al., 2014). These features imply that while the differentiation of laminar subtypes relies on the precise regulation of spatiotemporal expression and expression levels of the key genes, the procedure of neocortical neurogenesis and assembly is robust. Such an adaptable system would enable cells to respond to extrinsic cues provided within and outside the neocortex, which may underlie the significant cortical expansion during evolution.

### Future directions

Neocortical assembly is a highly intricate process that requires multiple layers of regulation in cell behavior at the progenitor and postmitotic cell stages. The emerging picture of neocortical assembly is that while the identities of neuronal subtypes are largely determined at birth, the mechanisms by which these neurons are navigated to their final positions involve cell type- and context-dependent combinatorial codes that enable their precise integration into the neocortical circuit. While the original finding indicated that neural stem cells undergo progressive restrictions in cell competence to sequentially produce the principal layer types (Frantz and McConnell, 1996; Desai and McConnell, 2000), the molecular logic underlying these subtype transitions has only begun to unravel over the past years. Importantly, these studies also provided new insights into how the timing and quantity of the production of each neuron subtype are controlled. While the appearance of RGCs and the elaboration of early preplate cells were likely the driving force of neocortical cytoarchitecture that enabled its tangential expansion during evolution (Pollard et al., 2006; Abellan and Medina, 2009), our current understanding of the mechanisms of neocortical assembly relies heavily on the regulatory molecules and their functions identified through mouse studies. However, in an evolutionary context, the timing of production and integration of each of the neuronal subtypes must be coordinated on a species-specific developmental time scale. This process is a particular challenge for gyrencephalic mammals with an enlarged cortex, which have increased gestational period, cell cycle or division modes. Growing evidence now demonstrates that the transitions between sequential layer subtypes utilize a regulatory system that integrates both intrinsic and extrinsic mechanisms. This system not only provides qualitative cues for the migration and integration of neurons at the correct timing but quantitatively calibrates the numbers of each subtype based on the presence of their counterparts. Such hierarchical transcriptional and intercellular network organization promotes the cost-effective production and wiring of neurons during development and evolution. Continuous efforts to decipher the molecular mechanisms of subtype-specific neuronal differentiation and their integration, would facilitate our understanding of the logic that balance between economical brain assembly and vulnerability to pathological conditions.

### Conflict of interest statement

The authors declare that the research was conducted in the absence of any commercial or financial relationships that could be construed as a potential conflict of interest.
